# Risk Factors for Seeking Medical Care Following Nirmatrelvir-Ritonavir (Paxlovid) Treatment for COVID-19: “Symptom Rebound”

**DOI:** 10.3390/v17060782

**Published:** 2025-05-29

**Authors:** Ashish Bhargava, Susan Szpunar, Mamta Sharma, Louis Saravolatz

**Affiliations:** Department of Medicine, Thomas Mackey Center for Infectious Disease Research, Henry Ford St. John Hospital, 19251 Mack Avenue, Suite 340, Grosse Pointe Woods, MI 48236, USA

**Keywords:** Paxlovid, nirmatrelvir–ritonavir, risk factors, predictors, COVID-19

## Abstract

Nirmatrelvir plus ritonavir (NPR) has been approved for treating mild to moderate COVID-19 in high-risk adults but concerns about rebound effects have limited its use. This study aimed to identify individuals at risk of seeking medical care among high-risk non-hospitalized patients treated with NPR from 1 January 2022 to 31 December 2022, at our institution. Our outcome variable was the composite of subsequent evaluation in the Emergency Department or inpatient admission within four weeks of their NPR treatment. Of 369 patients who received NPR treatment, the mean (SD) age was 59.3 (±13.8) years; 64% (236) were female, and 77.7% (281) were white. The incidence of the composite event was 6.8% (25/369). In multivariable logistic regression, factors for seeking medical care following NPR treatment were female sex (OR 4.6; 95% CI 1.4–15.3; *p* = 0.013), myocardial infarction (OR 4.1; 95% CI 1.4–11.8; *p* = 0.011), chronic lung disease (CLD) except asthma and chronic obstructive pulmonary disease (COPD) (OR = 3.9, 95% CI 1.1–13.5; *p* = 0.03), and diabetes mellitus with complications (OR 6.9; 95% CI 2.0–23.3; *p* = 0.002) while alcohol users (OR 0.39; 95% CI 0.2–0.9; *p* = 0.038) were less likely to seek medical care. Larger cohorts are necessary to further assess and confirm these risk factors.

## 1. Introduction

Paxlovid, a combination of the oral drugs nirmatrelvir plus ritonavir (NPR), has been approved by the US Food and Drug Administration for treating mild to moderate coronavirus disease 2019 (COVID-19) in high-risk adults [1]. The randomized placebo-controlled EPIC-HR study showed an 88.9% reduction in the relative risk of progression to hospitalization or death by day 28 for high-risk, non-hospitalized patients [2]. Despite its effectiveness, uptake has remained limited in COVID-19 patients because of concerns about rebound phenomena [3].

After the emergency use authorization for a five-day course of NPR for COVID-19 in December 2021, there were reports of signs or symptoms recurring, or a new positive viral test result after initial recovery from COVID-19 [4,5]. In May 2022, the US Centers for Disease Control and Prevention (CDC) issued an official health advisory regarding the potential for COVID-19 recurrence or “COVID-19 rebound” after NPR treatment [6]. Several retrospective studies showed that patients with mild-to-moderate COVID-19, treated with NPR, experienced a rebound rate ranging from 0.8% to 6.6% [7,8]. Given the concerns about the rebound phenomenon after NPR, EPIC-HR investigators retrospectively reviewed their phase 2–3 study data and noted viral rebound to be 2.3% in the NPR group and 1.7% in the placebo group [9]. Because of concerns about inaccurate incidence estimates, prospective studies were conducted to evaluate the occurrences of COVID-19 rebound. In the prospective cohort study, viral rebound incidences (14.3% vs. 9.3%) and symptom rebound incidences (18.9% vs. 7%) remained high in the NPR-treated group compared to untreated participants [10]. Given the high incidences of COVID-19 rebound, one in five healthcare professionals has concerns about prescribing NPR to older adults [3]. Factors associated with rebound or patients at risk of needing further medical attention are largely unknown. Identifying the patients who are more likely to experience a rebound after NPR treatment might help in the uptake of NPR or lessen the prescriber’s concerns about prescribing NPR within five days of a COVID-19 diagnosis.

The purpose of this study is to identify the risk factors for seeking medical care after receiving NPR treatment.

## 2. Methods

In this historical cohort study, we included high-risk, non-hospitalized, confirmed COVID-19 patients prescribed NPR from 1 January 2022 to 31 December 2022, at outpatient facilities associated with Ascension St. John Hospital. The initial identification of these patients started with a query of the outpatient medical record, Athena^®^, using COVID-19-related ICD-10 codes U07.1 and U07.2. J12.89, J20.8, J40, J22, J98.8 and J80 in conjunction with B97.29. From the Athena^®^ record, we identified patients who received a prescription for NPR. We reviewed the electronic medical records for demographic information and clinical details, including comorbid, tobacco, alcohol, and illicit drug use, vaccination status, confirmation of COVID-19 diagnosis, Emergency Department (ED) visits and/or hospitalizations within four weeks of NPR treatment. Trained dedicated research personnel contacted patients via telephone to confirm completion of the five-day NPR treatment and to assess if they needed urgent care or hospitalization. The study excluded patients without documentation of COVID-19 or who did not complete NPR treatment. The study was reviewed and approved by the Institutional Review Board. (Study number—RMI20220098).

## 3. Definitions

A “documented case of COVID-19” was defined as a positive screening test result with real-time reverse-transcriptase-polymerase-chain-reaction (RT-PCR) assay of nasopharyngeal swab specimens or documentation of home COVID-19 positive antigen test result prior to NPR treatment. Diabetic patients with complications had neuropathy, retinopathy, and nephropathy. “Immunosuppressed status” includes HIV positivity, neutropenia with absolute neutrophil counts less than 0.50 K/mcL at the time of hospitalization, receipt of steroids (equivalent to at least 15 mg of prednisone per day for at least seven days consecutively) at the time of hospitalization, receipt of chemotherapy or radiotherapy or anti-TNF [tumor necrosis factor]-α therapy in the past three months, and history of organ transplant. Paxlovid rebound was defined as the return of COVID-19 symptoms or a new positive viral test after the initial recovery.

## 4. Statistical Analysis

Descriptive statistics were calculated to characterize the study group. Continuous variables such as age were described as the mean with standard deviation or median with range or interquartile range, depending upon the normality of the distribution of the data. Categorical variables were described as frequency distributions. Our outcome variable was the composite of subsequent evaluation in the ED and/ or inpatient admission within four weeks of their NPR treatment. Univariable analysis was performed using Student’s *t*-test and chi-squared analysis. Multivariable analysis was performed using multiple logistic regression. All data were analyzed using SPSS v. 29.0, and a *p*-value less than 0.05 was considered to indicate statistical significance.

## 5. Results

Of 369 patients who received NPR treatment, the mean (SD) age was 59.3 (±13.8) years; 64% (236) were female, and 77.7% (281) were white. The cohort’s mean body mass index (BMI) was 31.3 ± 7.8 kg/m^2^. Tobacco use was noted in 43 (11.7%), alcohol use in 227 (62%), and substance use in 25 (6.8%). COVID-19 vaccination with at least one dose was noted in 311 (84.7%) patients. Common co-morbid conditions in patients were hypertension (53.7%), obesity (52.6%), chronic obstructive pulmonary disease (COPD) (27%), diabetes mellitus (DM) (20.5%), asthma (17.9%) and myocardial infarction (MI) (10.6%). A history of malignancy was found in 8.7% of patients, while immunosuppression was noted in 4.3% of patients.

The most common presenting symptoms among those seeking medical care after receiving NPR treatment were worsening shortness of breath (32%), cough (32%), body aches, and pains (32%). Four (16%) patients had gastrointestinal symptoms of nausea, vomiting, or diarrhea. Other presenting symptoms were headaches (12%), dizziness or lightheadedness (12%), chest pain (8%), sore throat (8%), abdominal pain (4%), dysuria (4%), bleeding, and coagulopathy (4%).

The incidence of the composite event was 6.8% (25/369). The risk of the composite event was higher in patients with a history of MI (28% vs. 9.3%), congestive heart failure (CHF) (12% vs. 2.3%), COPD (44% vs. 25.8%), chronic lung diseases (CLD) except asthma and COPD (20% vs. 5.4%) and DM with complication (24% vs. 4.1%), respectively (Table 1). Alcohol users were less likely to seek medical care following NPR (40% vs. 63.6%, respectively). The groups had no differences in BMI, tobacco use, or vaccination rates.

In multivariable logistic regression, factors associated with seeking medical care post-NPR were female sex (OR 4.6; 95% CI 1.4–15.3; *p* = 0.013), alcohol use (OR 0.39; 95% CI 0.2–0.9; *p* = 0.038), MI (OR 4.1; 95% CI 1.4–11.8; *p* = 0.011), DM with complications (OR 6.9; 95% CI 2.0–23.3; *p* = 0.002), and CLD (OR = 3.9, 95% CI 1.1–13.5; *p* = 0.03), at the time of NPR treatments (Table 2).

## 6. Discussion

According to our study, the independent risk factors for seeking medical care after receiving NPR treatment for COVID-19 were female sex, patients with a history of MI, CLD excluding asthma and COPD, and DM with complications. Interestingly, individuals who reported alcohol use were found to be less likely to seek medical care following the NPR treatment. Before our study, risk factors for symptom rebound following NPR therapy remained unknown, but factors associated with viral rebound assessing the longitudinal viral RT-PCR were reported [11]. In our study cohort, the incidence of subsequent evaluation in the ED and/or inpatient admission within four weeks of their NPR treatment was 6.8%. This finding was consistent with the previously reported rates of COVID-19 symptom rebound in retrospective studies [7,8]. In a prospective observational study, the incidence of symptom rebound was higher among participants treated with NPR (18.9%) than among those not treated (7.0%) with NPR [10]. Despite the high rebound rates observed after the drug treatment, the progression to severe disease and mortality remained uncommon in these studies.

The biological underpinning of this phenomenon is unclear, and its suggested causes are still a matter of debate. One observational study among six patients with rebound after NPR treatment showed a robust immune response with rising total and virus-specific T lymphocytes and biomarkers of T-cell activation suggesting an emerging immune response against residual viral antigens throughout the respiratory tract, likely reducing the risk for disease progression [12]. This proposed mechanism, however, fails to explain the findings in multiple studies, which have shown that persons with immunocompromising conditions are at higher odds of rebound among treated patients [13,14,15]. Another observational study found that individuals experiencing a rebound had a longer duration of infectious virus shedding (14 days) compared to those without a rebound (3 days) [13]. Genomic sequencing in this study, however, did not show any evidence of resistance-associated mutations, which aligns with findings from other studies [12,16]. The above viral dynamics and other possibilities of insufficient drug exposure by individual pharmacokinetics or inadequate duration of NPR treatment could be other etiological explanations for the rebound [16].

In our study, patients with a history of MI, CLD excluding patients with asthma and COPD, and diabetes with complications, were more likely to seek medical care following the NPR treatment. During the early phase of the pandemic and even after the development of COVID-19-directed therapies, patients with multiple underlying medical conditions were at risk of severe disease and poor outcomes [17,18,19,20,21,22]. Patients with CLD have structural pulmonary abnormalities and impaired immune defenses, putting them at risk for severe infection, including respiratory failure [19,23]. Some asthma treatments, such as dupilumab, reslizumab, and bevacizumab, may enhance immune responses to viral infections, including COVID-19, potentially reducing any additional lung injury [24]. In our study cohort, patients who reported having asthma had similar odds of seeking medical attention with NPR treatment, which could also vary based on the severity of asthma. Cardiac patients are susceptible to endothelial and vascular damage due to COVID-19, increasing the likelihood of acute coronary syndrome and blood clot formation, leading to a higher risk of seeking medical care [25]. People with diabetes are at higher risk of SARS-CoV-2 infection, and poor glycemic management entails an increased need for treatment and hospitalizations [26,27]. Diabetic patients exhibit dysregulated cytokine responses and impaired antiviral interferon responses, resulting in heightened inflammatory reactions. Hyperglycemia hinders the body’s immune defenses, affecting the function of white blood cells and lymphocytes, resulting in a weakened immune response and severe respiratory issues [28]. Patients with concomitant corticosteroids and NPR are also noted to have a higher likelihood of viral rebound [8]. Previous observations among patients experiencing rebound symptoms after NPR have highlighted the presence of multiple medical comorbid conditions [7,8].

In our study, more female patients sought medical care following NPR treatment. Another study reported a similar finding, with more females experiencing rebound symptoms after NPR treatment [29]. Women have a higher likelihood of being infected with COVID-19, while men have higher mortality rates from acute infection [30,31]. We have previously reported different risk factors for severe COVID-19 and in-hospital mortality among males and females [32]. Women exhibit stronger CD8+ T cell activity, increased CD4+ T cells, and heightened IgG antibody production [31,33]. This is protective in the early phase of COVID-19 but can prolong inflammation if consistently elevated [33]. Women produce higher antibody titers in response to the trivalent inactivated seasonal influenza vaccination and report more severe local and systemic side effects [34]. Additionally, women are generally more health-conscious and are more likely to seek medical attention than males.

In our study, patients who reported alcohol use were less likely to seek medical care following NPR treatment. A large prospective cohort study examining different types of alcoholic beverages and their association with a COVID-19 risk revealed that red wine, white wine, and champagne are linked to a reduced risk of COVID-19, while beer, cider, and spirits are associated with an increased risk [35]. Alcoholic beverages contain varying amounts of polyphenols, which have antioxidant properties [36]. They are particularly abundant in wines, and red wines have the highest concentrations of these phenolic compounds [36,37]. Another study found that consuming alcohol below the recommended guideline may reduce the risk of COVID-19 compared to non-drinkers [38]. Studies have shown that long-term alcohol abuse and acute binge drinking are associated with immunosuppression and increased susceptibility to both bacterial and viral infections [39,40,41].

Our study has several limitations: first, it was a single institution study. Second, it lacks virologic characterization, including the incidence of viral rebound. Third, we were unable to characterize the types of alcohol consumption and the amount of alcohol use around the patient’s COVID diagnosis and treatment. Fourth, we could not reliably determine the effect of the number of vaccine doses among the two groups due to the phone-based survey. Nevertheless, our study provides valuable insight into the factors contributing to the risk of seeking medical attention after NPR treatment for COVID-19.

In summary, independent risk factors for seeking medical care following NPR treatment for COVID-19 were female sex, MI, CLD, and DM with complications while alcohol users were less likely to seek medical care following the NPR treatment. Further detailed evaluation in larger cohorts is necessary to further assess and confirm these risk factors for COVID-19 rebound after NPR treatment.

## Figures and Tables

**Table 1:** Univariable analysis of risk factors for seeking medical care following nirmatrelvir–ritonavir (Paxlovid) treatment of COVID-19.

Characteristics	No ED/H Visit (*n* = 346) (%)	ED/H Visit (*n* = 25) (%)	*p* Value
Age in years (mean ± SD)	59.1 ± 13.6	62.2 ± 15.6	0.30
Sex			0.08
Male	128 (37.2)	5 (20.0)	
Female	216 (62.8)	20 (80.0)	
Race			0.7
Blacks	58 (17.3)	5 (7.9)	
Whites	263 (78.3)	18 (72.0)	
Others	15 (4.2)	2 (8.0)	
Body mass index (mean ± SD)	31.4 ± 7.82	30.3 ± 7.8	0.52
Current smoking	37 (10.8)	6 (24.0)	0.05
Alcohol use	217 (63.6)	10 (40.0)	0.02
Substance abuse	23 (6.7)	2 (8.0)	0.80
Vaccination	288 (84.2)	23 (92.0)	0.30
Myocardial infarction	32 (9.3)	7 (28.0)	0.003
Congestive heart failure	8 (2.3)	3 (12.0)	0.006
Hypertension	184 (53.3)	14 (56.0)	0.8
Asthma	60 (17.4)	6 (24.0)	0.41
COPD	89 (25.8)	11 (44.0)	0.05
CLD except asthma and COPD	15 (4.3)	5 (20.0)	<0.001
Connective tissue diseases	11 (3.2)	1 (4.0)	0.83
DM without complications	51 (14.8)	5 (20.0)	0.48
DM with complications	14 (4.1)	6 (24.0)	<0.001
Cerebrovascular disease	18 (5.2)	1 (4.0)	0.79
Metastatic solid tumor	1 (0.3)	1 (4.0)	0.02
Immunosuppressed status	15 (4.3)	1 (4.0)	0.93

Abbreviations: ED: emergency department; H: hospitalization; *n*: number; SD: standard deviation; CLD: chronic lung disease; COPD: chronic obstructive pulmonary disease; DM: diabetes mellitus.

**Table 2:** Multivariable analysis of risk factors for seeking medical care following nirmatrelvir–ritonavir (Paxlovid) treatment of COVID-19.

Variable	OR (95% CI)	*p* Value
Female sex	4.6 (1.4–15.3)	0.013
Alcohol use	0.4 (0.2–0.9)	0.038
Myocardial Infarction	4.1 (1.4–11.8)	0.011
DM with complication	6.9 (2.0–23.3)	0.002
CLD except asthma and COPD	3.9 (1.1–13.5)	0.03

Abbreviations: OR: odds ratio, CI: confidence interval, CLD: chronic lung disease, COPD: chronic obstructive pulmonary disease, DM: diabetes mellitus.

## Data Availability

The data set was gathered from the study site and is not accessible to the public.
